# Evaluation of an inflammation-based prognostic score (GPS) in patients with metastatic breast cancer

**DOI:** 10.1038/sj.bjc.6602922

**Published:** 2006-01-10

**Authors:** A M Al Murri, J M S Bartlett, P A Canney, J C Doughty, C Wilson, D C McMillan

**Affiliations:** 1 University Department of Surgery, Royal and Western Infirmaries, Glasgow, UK; 2 Beatson Oncology Centre, Western Infirmary, Glasgow, UK

**Keywords:** metastatic breast cancer, systemic inflammatory response, C-reactive protein, albumin, prognostic score, survival

## Abstract

Prediction of outcome in patients with metastatic breast cancer remains problematical. The present study evaluated the value of an inflammation-based score (Glasgow Prognostic Score, GPS) in patients with metastatic breast cancer. The GPS was constructed as follows: patients with both an elevated C-reactive protein (>10 mg l^−1^) and hypoalbuminaemia (<35 g l^−1^) were allocated a score of 2. Patients in whom only one or none of these biochemical abnormalities was present were allocated a score of 1 or 0, respectively. In total, 96 patients were studied. During follow-up 51 patients died of their cancer. On multivariate analysis of the GPS and treatment received, only the GPS (HR 2.26, 95% CI 1.45–3.52, *P*<0.001) remained significantly associated with cancer-specific survival. The presence of a systemic inflammatory response (the GPS) appears to be a useful indicator of poor outcome independent of treatment in patients with metastatic breast cancer.

Breast cancer is the second most common cause of cancer-related death among women in the Western world (Cancer Stats, www.cancerresearchuk.org; Jemal *et al*, 2003). Approximately, 10% of newly diagnosed breast cancer patients have locally advanced and/or metastatic disease at the time of presentation (Sant, 2001; Li *et al*, 2003). In addition, more than 40% of patients, who are diagnosed with early-stage breast carcinoma, will eventually experience later recurrence and/or metastatic disease (Early Breast Cancer Trialists' Collaborative Group, 1998).

Metastatic breast carcinoma exhibits a great deal of variability in its clinical presentation and behaviour. The prognosis is generally poor with a median overall survival of approximately 2–3 years (Ali *et al*, 2003; Bernard-Marty *et al*, 2004). Current therapies are palliative, aiming at improving or maintaining quality of life, controlling symptoms, and prolonging survival. Nevertheless, specific subgroups of patients exist for which, depending on the site of metastasis and treatment given, survival may range from a few months to several years (Nomura, 1998; Insa *et al*, 1999). This prognostic uncertainty has driven the search for well-standardised laboratory-based parameters, which have prognostic value.

Previous studies have established the prognostic importance of the systemic inflammatory response, as evidenced by an elevated circulating C-reactive protein concentration, in patients with advanced solid tumours (O'Gorman *et al*, 2000; Mahmoud and Rivera, 2002; Maltoni *et al*, 2005) including breast cancer (Albuquerque *et al*, 1995; Zhang and Adachi, 1999). Recently, we have shown that, using established cutoffs, a combination of an elevated C-reactive protein and hypoalbuminaemia, the Glasgow Prognostic score (GPS) has prognostic value, independent of stage and performance status, in patients with inoperable non-small-cell lung cancer (Forrest *et al*, 2003, 2004, 2005). However, there is no information on the prognostic value of this combination in patients with metastatic breast cancer.

The aim of the present study is to examine the relationship between the GPS and survival in patients with metastatic breast cancer.

## PATIENTS AND METHODS

Breast cancer patients presenting, with metastatic relapse, to a single oncology clinic in the Beatson Oncology Centre between February 2002 and November 2004 and who had a measurement of C-reactive protein and albumin undertaken were included in the study. At this time no patients showed clinical evidence of infection or other inflammatory conditions.

All patients had confirmed metastatic disease on the basis of either clinical findings or imaging. Patients were group according to whether they soft tissue and/or bone metastases, visceral metastases and visceral and soft tissue and/or bone metastases. Oestrogen receptor (ER) status was considered as positive when nuclear staining was seen in ⩾10% of cancer cells.

Patients who developed other malignancies, and patients with C-reactive protein measured during a course of taxane therapy were excluded from the study since they may produce a hypersensitivity reaction and elevated concentrations of interleukin-6 (Tsavaris *et al*, 2002; Williams *et al*, 2003), which is a primary mediator of C-reactive protein (Gabay and Kushner, 1999). In total, 96 out of 295 (33%) patients with metastatic disease were eligible for the study.

The study was approved by the Research Ethics Committee of the North Glasgow NHS Trust.

Routine laboratory measurements of C-reactive protein and albumin concentrations were carried out. The coefficient of variation was less than 5% as established by routine quality control procedures. The limit of detection of the assay is a C-reactive protein concentration of less than 6 mg l^−1^.

The GPS was constructed as previously described (Forrest *et al*, 2003). Briefly, patients with both an elevated C-reactive protein (>10 mg l^−1^) and hypoalbuminaemia (<35 g l^−1^) were allocated a score of 2. Patients in whom only one of these biochemical abnormalities was present were allocated a score of 1. Patients in whom neither of these abnormalities was present were allocated a score of 0.

### Statistics

Grouping of the variables was carried out using standard thresholds. Survival (cancer-specific) analysis was performed using the Cox proportional hazard model. Deaths up to 31st May 2005 have been included in the analysis. Multivariate survival analysis was performed using a stepwise backward procedure to derive a final model of the variables that had a significant independent relationship with survival. To remove a variable from the model, the corresponding *P*-value had to be greater than 0.10. Analysis was performed using SPSS software (SPSS Inc., Chicago, IL, USA).

## RESULTS

The baseline clinicopathological characteristics of the patients with metastatic breast cancer (*n*=96) are shown in Table 1. The majority of patients were over 50 years of age (78%), and received active treatment (96%). C-reactive protein and albumin concentrations were measured prior to systemic therapy in 35 patients and during systemic therapy in 61 patients. In all, 72 (75%) patients had not received cytotoxic chemotherapy for metastatic disease prior to the measurement of C-reactive protein and albumin concentrations.

In all, 92 (96%) patients received active treatment in the form of chemotherapy and/or endocrine therapy. In those patients in whom Her-2 status was assessed, 14 out of 47 patients were Her-2 positive and 11 of the 14 patients received Herceptin therapy. The majority of patients had C-reactive protein (53%) and albumin (94%) concentrations in the normal range; the GPS was elevated in 47% of patients. Of the six patients with hypoalbuminaemia, all had an elevated C-reactive protein concentration.

The minimum follow-up was 7 months; the median follow-up of the survivors was 16 months. During this period 51 patients died of their cancer. On univariate analysis, an elevated C-reactive protein (*P*<0.01), hypoalbuminaemia (*P*<0.05), the GPS (*P*<0.001) and treatment (*P*<0.05) were associated with poor cancer-specific survival. On multivariate analysis of the GPS and treatment, only the GPS (HR 2.26, 95% CI 1.45–3.52, *P*<0.001) remained significantly associated with cancer-specific survival.

**Figure 1:**
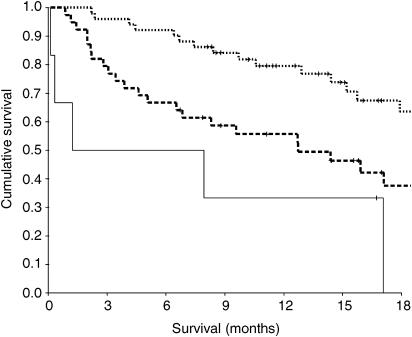
The relationship between an inflammation-based prognostic score (GPS, 0, 1, 2 from top to bottom) and survival in patients with metastatic breast cancer.

The relationship between clinicopathological characteristics and an inflammation-based prognostic score (GPS) in patients with metastatic breast cancer is shown in Table 2. The median survival in these patients was 24 months, 13 months and 1 month for a GPS of 0, 1 and 2, respectively (Figure 1).

## DISCUSSION

In the present study, a simple inflammation-based prognostic score (GPS) based on standard laboratory measurements of C-reactive protein and albumin, was an independent predictor of survival in patients with metastatic breast cancer. This work is consistent with previous work in patients with inoperable non-small-cell lung cancer (Forrest *et al*, 2003, 2004, 2005) and improves on the prediction of survival using an elevated C-reactive protein alone (Albuquerque *et al*, 1995; Zhang and Adachi, 1999). If these results are confirmed in larger studies, the GPS may be useful in the assessment of advanced breast cancer patients at diagnosis and the stratification of patients entering randomised trials.

In the present study, performance status was not recorded in the majority of patients. However, almost all the patients received active treatment and this would suggest that they had similar good performance status. Moreover, performance status is subjective (Ando *et al*, 2001) and the GPS has been shown to be a prognostic factor, independent of performance status in patients with inoperable non-small-cell lung cancer (Forrest *et al*, 2003, 2004). Other recognised prognostic factors, which have been shown to affect survival post relapse, such as ER status and disease free interval, did not reach statistical significance. This was probably due to the small number of patients in the present study. In contrast, an increasing GPS, (in particular 0 *vs* 1) was associated with a halving of survival. These results may point to the strength of the systemic inflammatory response (the GPS) in predicting survival in the patient with metastatic breast cancer.

It has been previously shown that, in patients with inoperable non-small-cell lung cancer at diagnosis, approximately three-quarters of patients had an abnormal GPS (1 or 2) and that these patients had a significantly poorer outcome (Forrest *et al*, 2003, 2004). It was of interest that, in the present study, although the proportion of patients with an abnormal GPS was less (47%), those metastatic breast cancer patients with a GPS of 2 had a similarly poorer survival. It may be that the prognostic value of the GPS is independent of tumour type in patients with advanced cancer (Elahi *et al*, 2004).

The mechanisms by which a systemic inflammatory response (the GPS) might impact on survival in advanced cancer patients are still not well defined. It may reflect the proinflammatory cytokine activity, in particular interleukin-6 (McKeown *et al*, 2004), which not only stimulates breast tumour growth (Kurebayashi, 2000), but also produce profound catabolic effects on host metabolism (McMillan *et al*, 1998; Kotler, 2000). In this way, the presence and magnitude of a chronic systemic inflammatory response may produce progressive nutritional and functional decline, ultimately resulting in reduced survival. Indeed, this concept is consistent with the observation in the present study that all patients with hypoalbuminemia had an elevated C-reactive protein concentration.

At the time of diagnosis, there are well-established prognostic factors on which to base the prediction of likely survival in cancer patients. In contrast, predicting survival of patients with advanced disease is more problematic. As a result, clinicians often overestimate survival (Christakis and Lamont, 2000; Glare *et al*, 2003). The results of the present study suggest that the GPS may be useful in the assessment of survival in patients with metastatic breast cancer. It is simple to use and based on routinely available, well-standardised measurements.

## Figures and Tables

**Table 1:** Clinicopathological characteristics in patients with metastatic breast cancer: univariate survival analysis

	**Patients** **(*n*=96)**	**Hazard ratio** **(95%CI)**	***P*-value**
Age (⩽50/>50years)	21/75	0.70 (0.38–1.31)	0.266
Prior cytotoxic chemotherapies			
(0/⩾1)	72/24	1.42 (0.76–2.65)	0.266
			
*Metastatic site*			
(Nonvisceral/visceral/both)	43/14/39	1.08 (0.79–1.48)	0.625
			
*Oestrogen receptor status*			
(positive/negative)	63/27	1.17 (0.65–2.12)	0.594
			
*Disease-free interval*			
(>2/⩽2 years)	67/29	1.16 (0.63–2.11)	0.633
			
*White cell count*			
(⩽11/>11 × 10^9^ l^−1^)	74/3	1.13 (0.15–8.34)	0.904
			
*Haemoglobin*			
(⩾12/<12 g dl^−1^)	46/30	1.75 (0.90–3.42)	0.1
			
*C-reactive protein*			
(⩽10/>10 mg l^−1^)	51/45	2.50 (1.40–4.48)	0.002
			
*Albumin concentration*			
(⩾35/<35 g l^−1^)	90/6	3.41 (1.33–8.72)	0.011
			
*GPS*			
(0/1/2)	51/39/6	2.26 (1.45–3.52)	<0.001
			
*Treatment*			
(Herceptin/active/supportive)	11/81/4	2.29 (1.02–5.19)	0.046

**Table 2:** The relationship between clinicopathological characteristics and an inflammation-based prognostic score (GPS) in patients with metastatic breast cancer

	**GPS 0 (*n*=51)**	**GPS 1 (*n*=39)**	**GPS 2 (*n*=6)**	***P*-value**
Age (⩽50/>50years)	10/41	10/29	1/5	0.751
Prior cytotoxic chemotherapies				
(0/⩾1)	36/15	32/7	4/2	0.409
				
*Metastatic site*				
(Nonvisceral/visceral/both)	24/9/18	16/5/18	3/0/3	0.684
				
*Oestrogen receptor status*				
(positive/negative)	27/18	30/9	6/0	0.061
				
*Disease-free interval*				
(>2/⩽2 years)	35/16	27/12	5/1	0.756
White cell count				
(⩽11/>11 × 10^9^ l^−1^)	40/1	28/2	6/0	0.58
				
*Haemoglobin*				
(⩾12/<12 g dl^−1^)	26/14	18/12	2/4	0.334
				
*Treatment*				
(Herceptin/active/supportive)	9/42/0	2/33/4	0/6/0	0.044
				
Survival (months)	23.8	12.7	1.2	
	(20.2–27.5)	(5.1–20.3)	(0.7–10.4)	<0.001
